# De-MSI: A Deep Learning-Based Data Denoising Method to Enhance Mass Spectrometry Imaging by Leveraging the Chemical Prior Knowledge

**DOI:** 10.1021/acs.analchem.5c02946

**Published:** 2025-09-08

**Authors:** Lei Guo, Chengyi Xie, Xin Diao, Thomas Ka Yam Lam, Yanhui Zhong, Yanyan Chen, Jingjing Xu, Xiangnan Xu, Xiangyu Zhu, Zhuang Xiong, Shangyi Luo, Jianing Wang, Jiyang Dong, Zongwei Cai

**Affiliations:** † Interdisciplinary Institute for Medical Engineering, 12423 Fuzhou University, Fuzhou 350108, China; ‡ State Key Laboratory of Environmental and Biological Analysis, 26679 Hong Kong Baptist University, Hong Kong SAR 999077, China; § Department of Electronic Science, National Institute for Data Science in Health and Medicine, 12466 Xiamen University, Xiamen 36100, China; ∥ School of Business and Economics, 9373 Humboldt-Universitat zu Berlin, Berlin 10099, Germany; ⊥ School of Marine Science and Engineering, Hainan University, Haikou 570228, China; # College of Science, Eastern Institute of Technology, Ningbo 315200, China

## Abstract

Mass spectrometry imaging (MSI) is a label-free technique that enables the visualization of the spatial distribution of thousands of ions within biosamples. Data denoising is the computational strategy aimed at enhancing the MSI data quality, providing an effective alternative to experimental methods. However, due to the complex noise pattern inherent in MSI data and the difficulty in obtaining ground truth from noise-free data, achieving reliable denoised images remains challenging. In this study, we introduce De-MSI, a novel deep learning-based method specifically developed for denoising MSI data without ground truth. The core concept of De-MSI involves constructing the reliable training data set by leveraging prior knowledge of mass spectrometry from the noisy MSI data, followed by training a deep neural network to improve the data quality by removing the noise from the original images. De-MSI has demonstrated superior performance in improving data quality over the commonly used methods when applied to MALDI-acquired mouse fetus data sets on visual inspection. Quantitative evaluations further confirm its superiority, with De-MSI achieving a mean PSNR of 18.93 and a mean SSIM of 0.74 across all ion images. The ability of De-MSI to enhance data quality in high-resolution MSI data sets is confirmed using the mouse brain data set at a pixel size of 5 μm. Additionally, its application to denoise rat brain data sets using the DESI technique showcases its adaptability across different ionization methods. The proposed model holds significant promise as a vital tool for the efficient analysis and interpretation of MSI data.

## Graphical abstract

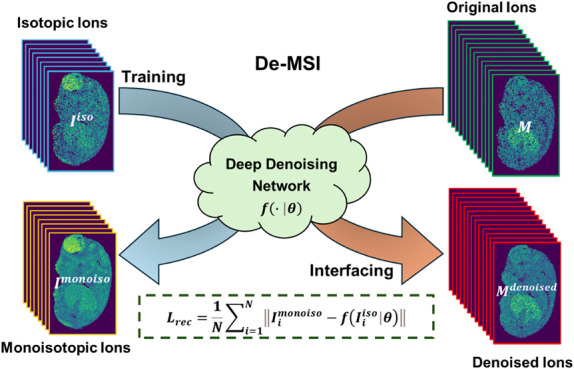

## Introduction

Mass spectrometry imaging (MSI) is a label-free imaging technique that enables capturing the spatial distribution of biomolecules within tissues with high sensitivity and specificity.^1,2^ It has been a key analytical chemistry technique with significant applications in drug development,^3^ environmental science,^4^ and clinical research.^5^ In general, a typical MSI experiment produces an extensive array of spatially resolved mass spectra, each containing thousands of distinct mass-to-charge (*m*/*z*) features. Although there is rich biologically relevant signal in MSI data, the noise introduced from data acquisition poses a challenge for effective mining and interpretation of MSI data.^6,7^ Improving the signal-to-noise ratio (SNR) for MSI data has become a crucial focus within MSI research, which would further expand its applicability in biomedical investigations.

The methods adopted to generate MSI data with high SNR are either an experimental strategy or a computational strategy. The experimental strategy typically involves optimizing sample preparation and instrumental parameters, which often requires refined sample pretreatment and fine-tuning of instrument settings to align with the specific properties of the sample and experimental conditions. Consequently, the experimental strategy can be time-consuming and demands experienced technicians to achieve the desired MSI data quality.^8^ On the other hand, the computational strategy applies the data denoising method directly to unideal MSI data to reduce noise while preserving meaningful biological signals.^9–12^ Since it does not require repeated experiments and can further enhance the quality of experimental data, the computational strategy offers an effective alternative to traditional experimental strategies and has emerged as a prominent area of research.

Recently, several computational strategies have been developed for data denoising, typically relying on specific mathematical assumptions concerning the data distribution of MSI data sets. For example, the Wavelet denoising assumes that the high-dimensional MSI data adheres to a Gaussian distribution, employing thresholding to remove noise in the high-frequency domain to enhance the data quality.^12^ Data filtering-based techniques, such as Gaussian filtering, reconstruct the signal at each data point by taking the spatial adjacent data point into consideration, based on the assumption that signal intensities of data points are spatially similar but the noise is random.^9^ However, it is well understood that noise within MSI data set is highly heterogeneous, with different noise sources exhibiting completely distinct characteristics across the data set.^6^ These variations may not be adequately addressed by a specific assumption, making existing methods to produce denoised images significantly deviate from the noise-free data.

Supervised deep learning leverages the data-driven strategy,^13,14^ which allows us to develop an alternative method for denoising MSI data without relying on predefined assumptions. Typically, it involves constructing a reliable training data set consisting of pairs of noisy data with low SNR and their corresponding noise-free data serving as the ground truth. The constructed data set is then used to train a deep denoising network to enhance the quality of unseen testing data, which have been extensively utilized in the denoising tasks of natural and optical imaging.^15,16^ However, different from the other imaging techniques, obtaining noise-free MSI data presents significant challenges, which means there is no completely reliable training data set to optimize the parameter of a deep denoising network. As a result, the existing supervised deep learning methods cannot be directly applied to denoise the MSI data.

Despite the challenges in obtaining completely noise-free MSI data, the training data set can be constructed based on the chemical prior knowledge from mass spectrometry. Specifically, the MSI technique typically captures redundant information, i.e., both monoisotopic ions and their isotopic variants are captured for a given molecule. In theory, the signal intensities of isotopic ions are expected to display spatial distributions identical to those of their monoisotopic counterparts. However, in practical applications, isotopic ions often display a higher incidence of missing values and outliers compared with monoisotopic ions, as illustrated in Figure S1. It is primarily due to their inherently lower intensity or unideal experimental conditions in real-world systems. Considering the natural isotopic abundance, the degradation of signal from monoisotopic ions to isotopic ions is the intrinsic property of most ions within the MSI data. Consequently, a training data set can be constructed where isotopic ions serve as input data, while monoisotopic ions provide pseudoground truth, facilitating the training of a deep neural network to enhance MSI data denoising.

In this study, we present De-MSI, a novel deep learning-based method for MSI data denoising. The method involves two stages: First, pseudoground truth is derived based on chemical prior knowledge from mass spectrometry. It involves identifying and pairing isotopic ions with their monoisotopic counterparts to establish a training data set. Second, the constructed training data set is used to train the deep denoising network, and then all original ion images are fed into the trained network to interface the final denoised images. The application of De-MSI to the MSI data set of a mouse fetus demonstrates that De-MSI surpasses commonly used methods through both visual inspection and quantitative evaluation. In the mouse brain data set, acquired at a pixel size of 5 μm, De-MSI effectively mitigates the pixel loss often associated with high-resolution MSI analyses. Additionally, we also perform the De-MSI on the rat brain data set analyzed using DESI-MSI, and the results highlight the versatility of the proposed De-MSI across various ionization sources. The proposed De-MSI is expected to become a widely used preprocessing tool for MSI data, facilitating large-scale analysis and interpretation.

## Materials

### Sample Preparation and Data Acquisition

All animal experiments conducted in this study were approved by the Committee on the Use of Human and Animal Subjects in Teaching and Research at Hong Kong Baptist University, ensuring compliance with ethical standards. Three representative MSI data sets are generated in-house to comprehensively evaluate the performance of our proposed De-MSI method: a mouse fetus analyzed using MALDI-MSI at a pixel size of 100 μm, a mouse brain analyzed using MALDI-MSI at 5 μm, and a rat brain analyzed using DESI-MSI at 100 μm. Detailed protocols for sample preparation and data acquisition are provided in Material S1. Subsequently, the MSI data are exported for further analysis.

### Data Preprocessing

Data preprocessing, including peak detection, peak alignment, peak filtering, peak pooling, hotspot elimination, and normalization, is conducted to generate ion images from exported MSI data. Specifically, the tasks of peak detection and peak alignment are accomplished using the SCiLs Lab (Bruker Company, Germany). The filtering, pooling, and total ion current (TIC) normalization processes are managed through custom Python scripts, as detailed in our previous work.^17^ Subsequently, the exported MSI data is transformed into a three-dimensional matrix, denoted as *M*_*X*×*Y*×*H*_. Here, *X* and *Y* represent the horizontal and vertical pixel counts, while *H* signifies the number of ion images corresponding to the *m*/*z* bins.

Additionally, signals in each ion image that exceed 99% of the maximum intensity are identified as hotspots and truncated to minimize their impact on visual analysis. The signal intensities within each ion image are then normalized to a [0, 1] range by dividing each intensity by the maximum signal intensity of that ion. The statistics for the three preprocessed MSI data sets are presented in Table S1.

## Methods

The workflow of the proposed De-MSI for MSI data denoising is illustrated in Figure 1. It consists of two stages: first, a training data set is constructed by leveraging the prior chemical knowledge from mass spectrometry; second, a denoising network is trained and utilized to perform inference on the original ion images, resulting in the denoised images. The proposed De-MSI can generate denoised MSI images without the requirement of ground truth data.

**Figure 1:**
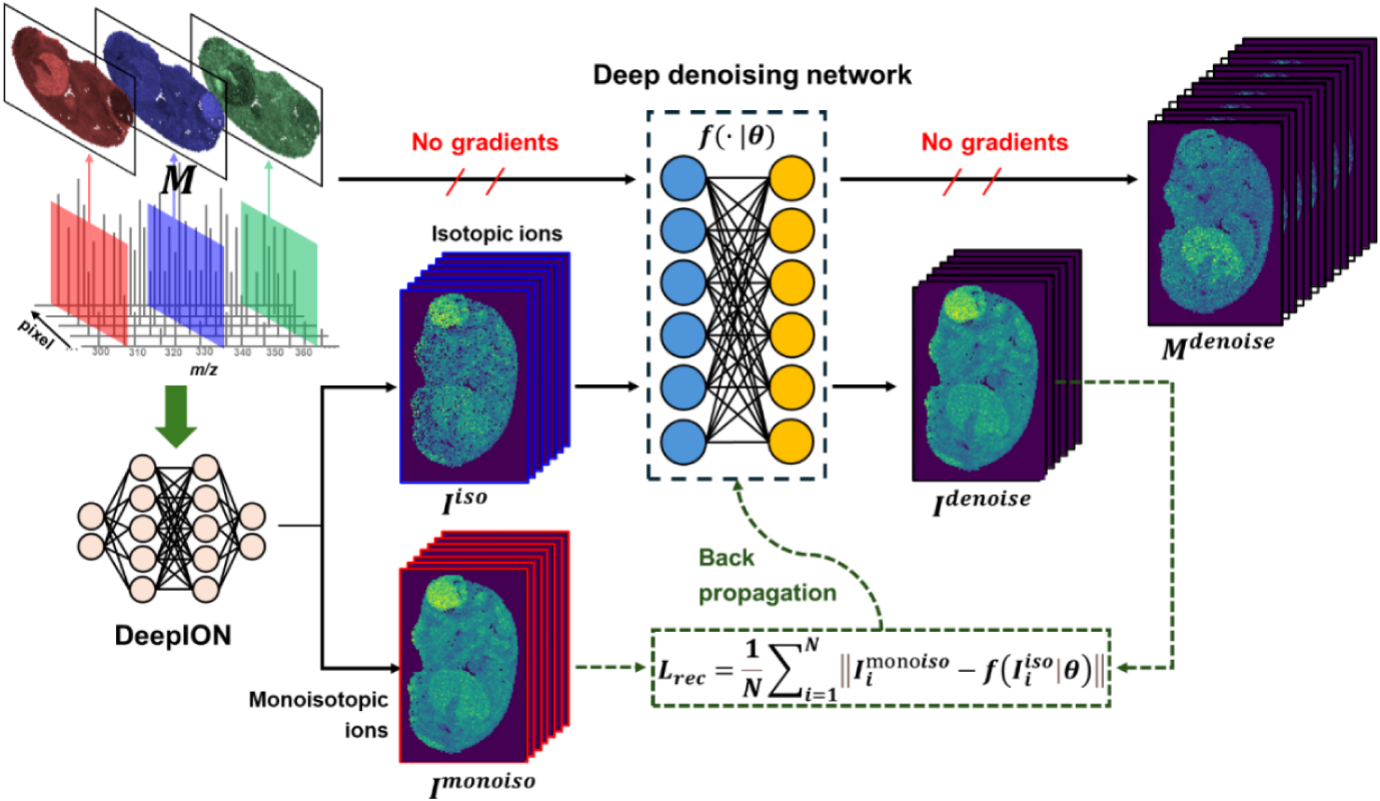
Overflow of the proposed De-MSI for MSI denoising. Initially, the pairs of isotopic ions *I* ^iso^ and monoisotopic ions *I* ^monoiso^ are identified using the presented DeepION or other established tools. Subsequently, *I* ^iso^ is processed through the deep denoising network, denoted as f(·|θ) , to produce the denoised images *I* ^denised^. The model optimization involves minimizing the reconstruction loss *L*_rec_, calculated as the mean absolute error between the output of the deep denoising network *I* ^denoised^ and the target *I* ^monoiso^. After training, the original MSI data *M* is input into the trained deep denoising network f(·|θ) to interface the final denoised output *M* ^denoised^.

### Workflow of Proposed De-MSI

In the first stage, our developed tool DeepION^17^ with ISO mode is employed to identify pairs of isotopic ions *I* ^iso^ and the corresponding monoisotopic ions *I* ^monoiso^ from preprocessed MSI data. Further details can be found in Material S2. Additionally, alternative tools like METASPACE^18^ and rMSI^19^ can also effectively perform the pair of monotopic and isotopic ion identifications, facilitating the construction of a reliable training data set. The pairs of isotopic ions and monoisotopic ions are then used to construct the training data set {*I* ^iso^,*I* ^monoiso^}.

In the second stage, the *I* ^iso^ and *I* ^monoiso^ are used as the input and the target to train the deep denoising network f(·|θ) . The loss function for f(·|θ) is the reconstruction term *L*_rec_ that calculates the mean absolute error (MAE) between the output $f\left(I^{\mathrm{iso}}|\theta \right)$ and the target *I* ^monoiso^, as follows:

1 $$L_{\mathrm{rec}}=\frac{1}{N}\overset{N}{\underset{i=1}{\sum }}|I_{i}^{\mathrm{monoiso}}-f\left(I_{i}^{\mathrm{iso}}|\theta \right)|$$

where θ is the parameters of the deep denoising network, *N* is the number of monoisotopic and isotopic ion pairs. After training the network f(·|θ) , the original preprocessed data *M* are then fed into the network f(·|θ) to interface the corresponding denoised data *M* ^denoised^.

### Model Architecture of Deep Denoising Network

With the availability of pseudoground truth data, the task of MSI data denoising can be adeptly tackled using a supervised deep learning model. The U-Net architecture is selected here for the construction of a deep denoising network f(·|θ) due to its efficient feature extraction, simplicity in implementation, and demonstrated effectiveness in medical imaging tasks with limited ground truth data.^20,21^ In this study, the model architecture of the U-Net framework is shown in Figure 2. Specifically, the U-Net architecture utilizes a pairwise encoder-decoder block to capture both local and global spatial information from isotopic ion images, enabling the generation of denoised ion images that closely resemble pseudoground truth, i.e., the corresponding monoisotopic ion image. The encoder consists of 2-dimensional convolutional neural network (2D CNN) layers with a 3 × 3 kernel size, followed by ReLU activations, progressively increasing feature channels from 64 to 1024, and includes downsampling via max-pooling. The decoder mirrors this structure, performing up-sampling with 3 × 3 2D CNN layers and bilinear up-sampling, reducing dimensions from 1024 to 64. A key feature of the U-Net architecture is its use of skip connections via copy-and-concatenate operations, which integrate high-resolution features from the encoder with the up-sampling layers of the decoder. It enhances the ability of the network to localize and recover details, making it highly effective in learning the signal patterns in MSI data.

**Figure 2:**
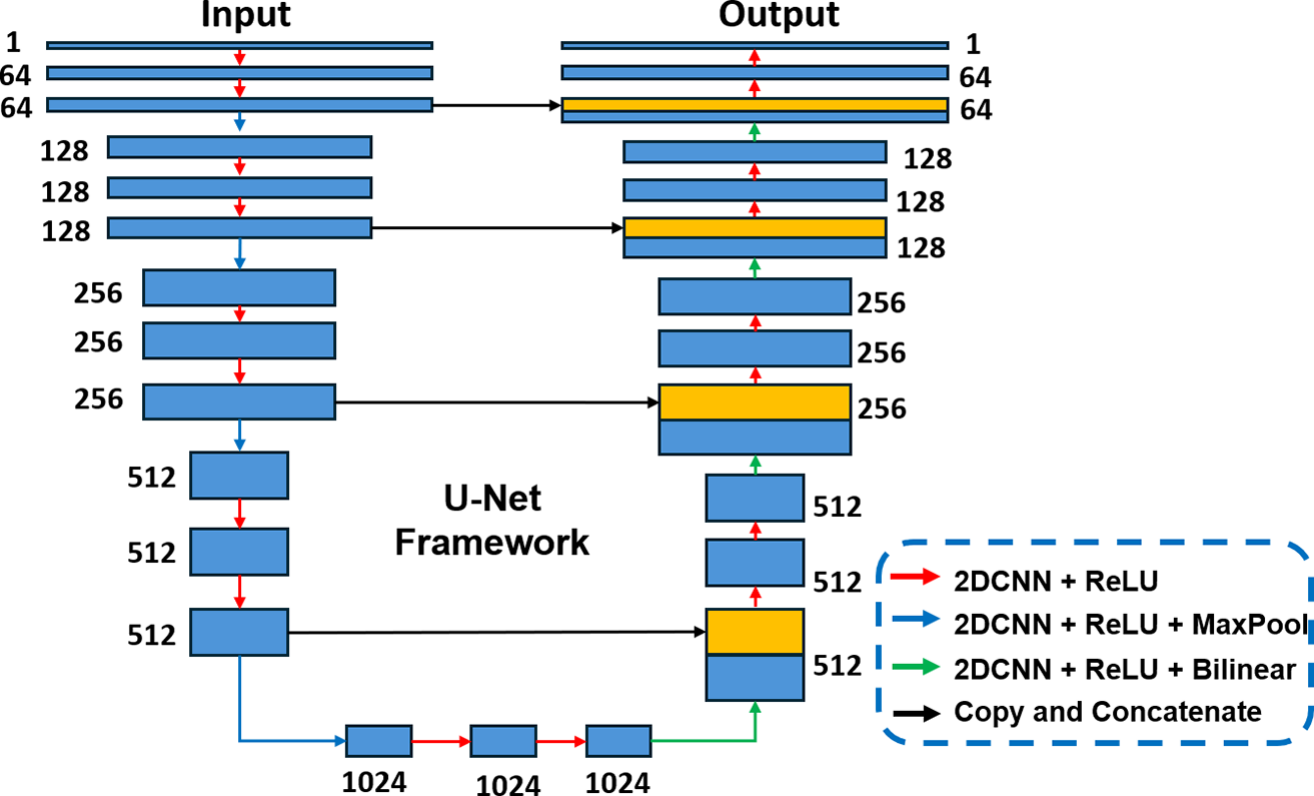
Model architecture of the U-Net framework. Each blue box represents a multichannel feature map, with the channel number indicated beside the box. Yellow boxes represent the copied feature maps. The arrows of different colors represent distinct operations, where the red indicates the operations of a 2D CNN followed by ReLU activation, blue represents a 2D CNN followed by ReLU activation and max pooling, green denotes a 2D CNN followed by ReLU activation and bilinear upsampling, and black illustrates the copy and concatenate operations.

### Model Implementation

The Adam optimizer is utilized to train the deep denoising network f(·|θ) , using a learning rate of 0.005, with the momentum parameters set to their default values.^22^ The adopted deep denoising network utilizes a lightweight U-Net architecture, which maintains a consistent set of parameters across inputs of varying shapes; in this study, the total number of parameters is approximately 310.4M. Initialization parameters for the De-MSI model are drawn from a normal distribution *N*(0,0.02). The batch size is set to 256. The model undergoes 20,000 epochs during training. The De-MSI model is implemented in Python using the PyTorch library, and training is conducted on a workstation equipped with an NVIDIA GTX 4090 GPU.

### Model Evaluation

The performance of the De-MSI is evaluated using both visual inspection and quantitative evaluation. Visual inspection utilizes prior information from optical imaging and common sense to assess denoised images, a method commonly employed in previous studies.^23,24^ Potential ions within the regions of interest are screened, and selected ions are manually annotated by matching their accurate masses to known metabolites in the LIPID MAPS database by applying a 10 ppm mass tolerance. Due to the challenge of obtaining noise-free data, quantitative evaluation involves adding simulated noise to the original ion images, with the original images serving as the ground truth, as reported in previous studies.^25^ Here, Poisson noise and random missing data are employed to simulate the noise typically generated by mass analyzers,^26–28^ as follows: (1) For each pixel in the ion image, a random intensity is generated to replace the original intensity, following a Poisson distribution with the pixel value as the mean. It is implemented using the “poisson.rvs” function from the SciPy Python package; (2) Randomly set 20% of the pixel signals in each ion image to zero. Then, two commonly used metrices, peak signal-to-noise ratio (PSNR)^29^ and structure similarity index measure (SSIM),^30^ are adopted to evaluate the performance at a quantitative level. Details of the quantitative evaluation are described in Material S3.

## Results and Discussion

### De-MSI Outperforms Other Methods on Visual Inspection and Quantitative Evaluation

The proposed De-MSI is used to conduct MSI denoising on the mouse fetus data set to enhance data quality. Representative isotopic-monoisotopic ion pairs from the training data are displayed in Figure S2. The commonly used techniques, such as Gaussian filtering and Wavelet denoising, are applied for comparative analysis. In the optical image, six organs are clearly identifiable: the brain, heart, cartilage, liver, small intestine, and kidney, as shown in Figure 3a. Two monoisotopic ion images, corresponding to *m*/*z* 603.1546 (unknown) and *m*/*z* 909.5437 (PI 40:6), are presented for comparison in Figure 3b,c. The results reveal that the ion with *m*/*z* 603.1546 exhibits high expression in the brain and cartilage regions, while the ion with *m*/*z* 909.5437 is highly expressed in the liver region. It is well established that metabolites tend to exhibit similar intensity among spatially adjacent pixels within each organ. However, due to the impact of noise, the original ion images exhibit missing intensity and anomalous expression, resulting in discontinuous ion appearance within the organ. Although noise reduction techniques such as Gaussian filtering and Wavelet denoising are applied, they both lead to the loss of high-frequency information, causing textures and boundaries to become blurred. Furthermore, the implementation of a window-based spatial information reconstruction strategy results in a noticeable degradation of resolution in the denoised outcomes. The proposed De-MSI utilizes the U-Net architecture, enabling effective extraction of both low-frequency and high-frequency information from MSI data. By training the U-Net architecture using pseudoground truth derived from the chemical prior knowledge from mass spectrometry, De-MSI produces the denoised images with enhanced data quantity compared to the commonly used methods on visual inspection. Additional denoised monoisotopic ion images produced by the De-MSI method are displayed in Figure S3. The running time for denoising with the De-MSI model is influenced by both the input data dimensionality and the selected batch size. Under the implementation settings of this study, the model requires 5.13 h for training and achieves an inference time of 1.92 s to denoise 586 ion images within the mouse fetus data set, both of which are suitable for offline denoising tasks. These results demonstrate the efficacy of the proposed De-MSI method in denoising MSI data.

**Figure 3:**
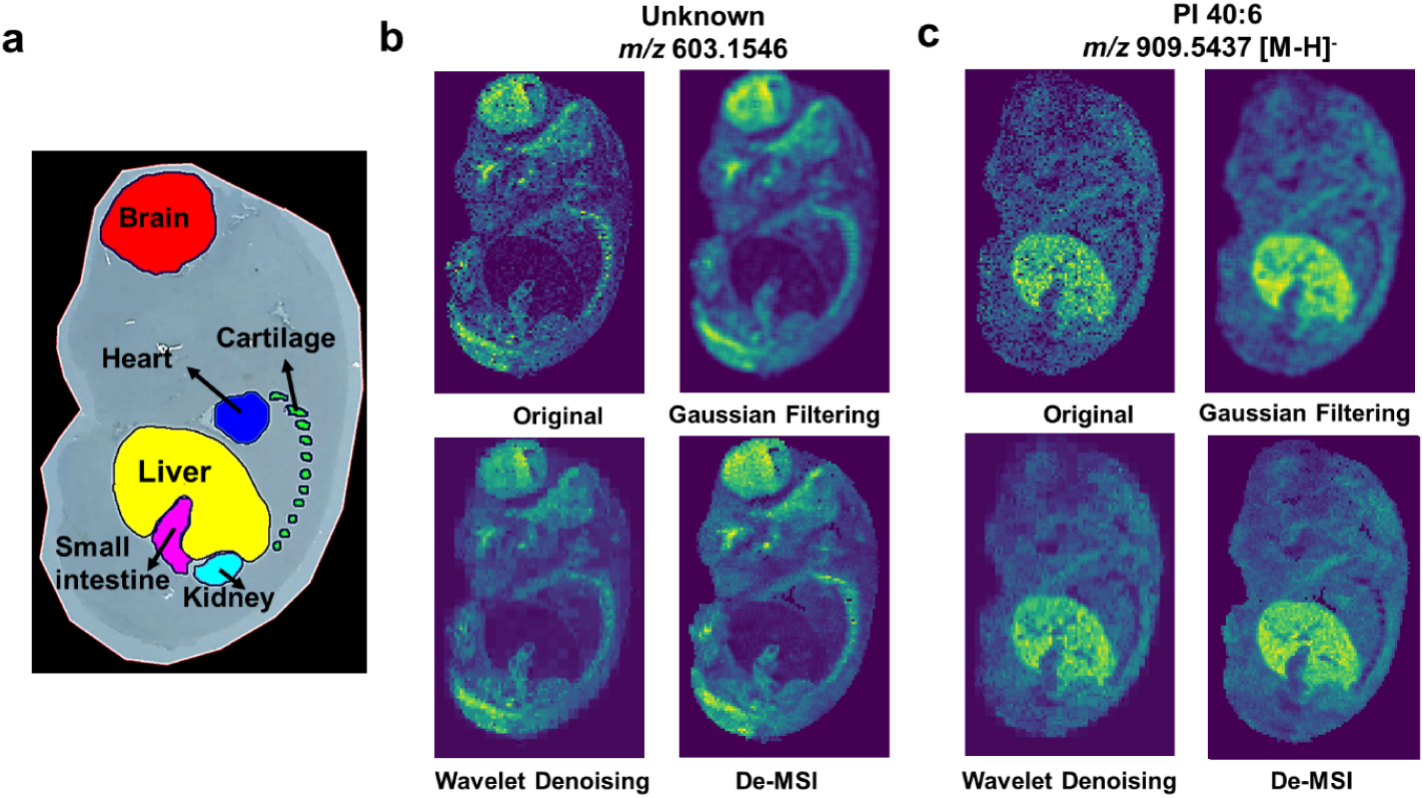
Comparison of De-MSI with other data denoising methods on the MSI dataset of a mouse fetus using visual inspection. (a) Optical imaging, with the brain, heart, cartilage, liver, small intestine, and kidney highlighted in red, blue, green, yellow, pink, and light blue, respectively. Spatial distribution of ion images for (b) *m*/*z* 603.1546, (c) *m*/*z* 909.5437, and the denoised image generated from Gaussian filtering, Wavelet denoising, and the proposed De-MSI method, respectively.

In addition, two metrics, PSNR and SSIM, are employed to quantitatively assess the performance of De-MSI in comparison with other methods, as illustrated in Figure 4. The simulated data, created by introducing Poisson noise and random missing elements to the original ion images, serves as the input noisy data, while the original image is used as the ground truth. Higher values of PSNR and SSIM between the denoised simulated image and the original image indicate superior performance of the denoising method. The results show that Gaussian filtering achieves a mean PSNR of 17.61 ± 1.62 and a mean SSIM of 0.61 ± 0.04, while Wavelet denoising results in a mean PSNR of 16.07 ± 1.54 and a mean SSIM of 0.50 ± 0.04. The proposed De-MSI achieves the highest performance, with a mean PSNR of 18.93 ± 1.87 and a mean SSIM of 0.74 ± 0.05, respectively. Figure S4 gives the spatial distribution of the original ion image, the noisy image, and the denoised images generated using various methods at *m*/*z* 279.2268, *m*/*z* 312.0643, *m*/*z* 415.2181, and *m*/*z* 726.5290. These comparative results further highlight the superior performance of the proposed De-MSI method on visual inspection. By leveraging prior knowledge of mass spectrometry from the noisy MSI data, the proposed De-MSI method effectively addresses outliers and missing values, achieving superior denoising results compared with commonly used methods.

**Figure 4:**
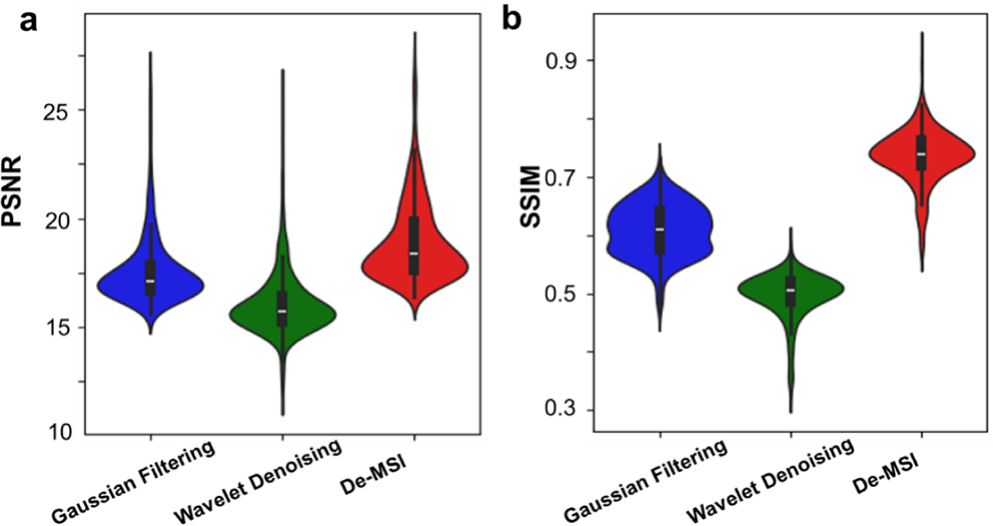
Comparison of PSNR and SSIM of De-MSI with other data denoising methods using quantitative evaluation. (a) PSNR. (b) SSIM.

### De-MSI Reduces the Noise of High-Resolution MSI Data

Recent advancements in mass spectrometry imaging have concentrated on achieving higher spatial resolution, which has markedly improved its utility in single-cell analysis.^31–33^ However, these advancements come with challenges, including decreased SNR due to a smaller sampling area, which complicates analysis and interpretation. To illustrate the effectiveness of De-MSI in high-resolution MSI data, the proposed De-MSI is performed on the data set of a mouse brain acquired from MALDI-MSI with 5 μm pixel size, as shown in Figure 5. Representative isotopic-monoisotopic ion pairs from the training data are displayed in Figure S5. Two boxed regions are zoomed in to the right panel for comparison between the original image and the denoised image. Here, seven regions, including the granule cell layer (DG-sg), stratum lacunosum-moleculare (CAslm), pyramidal layer (CA1sp), corpus callosum (CC), primary somatosensory area (SSp), caudoputamen (CP), and lateral dorsal nucleus of thalamus (LD), are identified from the optical image based on the previous works,^34^ as shown in Figure S6. The monoisotopic ion at *m*/*z* 309.2799 (FA 20:1) exhibits high expression in the CC region, whereas the monoisotopic ions at *m*/*z* 793.5534 (Unknown) and *m*/*z* 865.5030 (PG 44:12) show high expression in the DG-sg, CP, and CA1sp regions. Additionally, the monoisotopic ion at *m*/*z* 881.5183 (PI 38:6) is highly expressed in the DG-sg, CA1sp, and CAslm regions. Nevertheless, the improvement in spatial resolution is associated with a compromise in data quality, predominantly arising from the diminished molecular sampling per pixel and the intrinsic sensitivity constraints of the instrumentation at elevated resolutions. Using the proposed De-MSI method, a large number of missing values are imputed and outliers are reconstructed, resulting in subregions that become more continuous while maintaining sharp border definition. Figure S7 presents the comparative analysis of mean spectra between the original and denoised data, demonstrating that the proposed De-MSI model effectively preserves the integrity of the authentic signals without introducing artifacts that could compromise downstream analyses. In contrast, both Gaussian filtering and Wavelet denoising tend to produce oversmoothing results, resulting in the loss of high-frequency information, as shown in Figure S8. Other monoisotopic ions that are colocalized with the major subregions within the mouse brain are displayed in Figure S9. These results demonstrate that the proposed De-MSI can effectively mitigate the decrease of sensitivity often associated with high-resolution MSI analyses.

**Figure 5:**
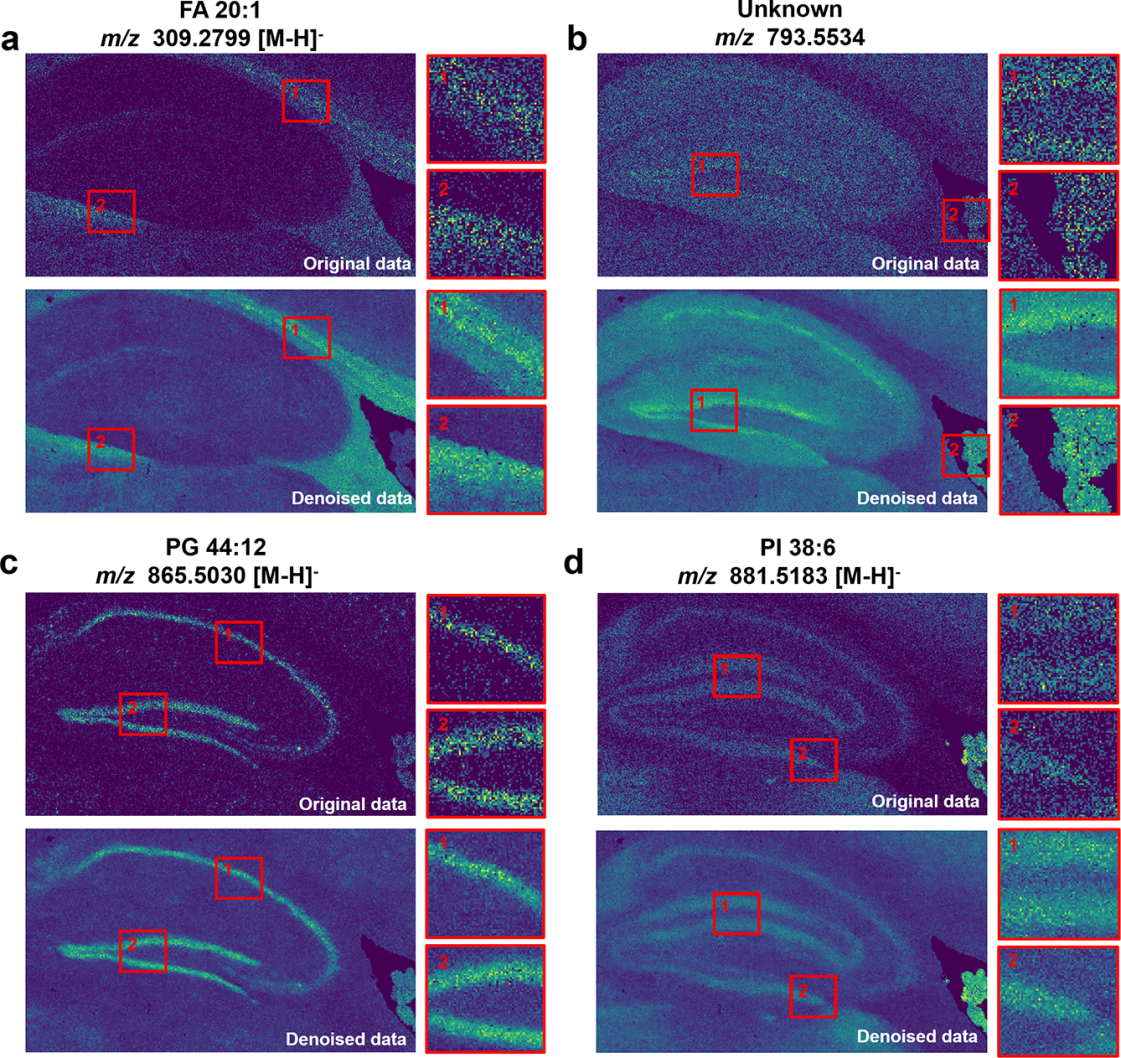
Comparison of original and denoised images from the MSI data set of a mouse brain, acquired via MALDI-MSI at a pixel size of 5 μm. (a) *m*/*z* 309.2799; (b) *m*/*z* 793.5534; (c) *m*/*z* 865.5030; (d) *m*/*z* 881.5183.

### De-MSI Improves the DESI-MSI Data Quality

To demonstrate the general applicability of the proposed De-MSI method, we applied it to a rat brain MSI data set obtained using the DESI-MSI technique. Here, we perform De-MSI on two representative monoisotopic ions, *m*/*z* 306.0766 (Glutathione) and *m*/*z* 760.5140 (PS 34:1), as illustrated in Figure 6. Representative isotopic-monoisotopic ion pairs from the training data are displayed in Figure S10. In the original ion image, the high expression levels of Glutathione are detected at the locations corresponding to the tail end of the CC region and the temporal pole (TP) region, while PS 34:1 demonstrates high expression in the cerebellar medulla (CM) region and the hippocampus (HP) region. Nevertheless, owing to surface irregularities in desorption and variations in ionization efficiency in electrospray during DESI-MSI data acquisition, these ions display spatial discontinuities within these regions. Using the proposed De-MSI method for data denoising, the results achieve greater denoised results on visual inspection. In addition, we also plot the intensity differences of the ion image at *m*/*z* 306.0766 and *m*/*z* 760.5140 relative to spatial distance. It is found that the denoised image exhibits lower intensity differences than the original image at varying distances, aligning with the common sense that identical subregions in the brain possess similar molecular profiles.^35^ Other spatial distributions of original ion images and denoised images within the MSI data set of the rat brain acquired using DESI-MSI are shown in Figure S11. These results show the efficacy of the proposed De-MSI for denoising MSI data acquired using DESI-MSI, demonstrating its versatility and applicability across various ionization sources.

**Figure 6:**
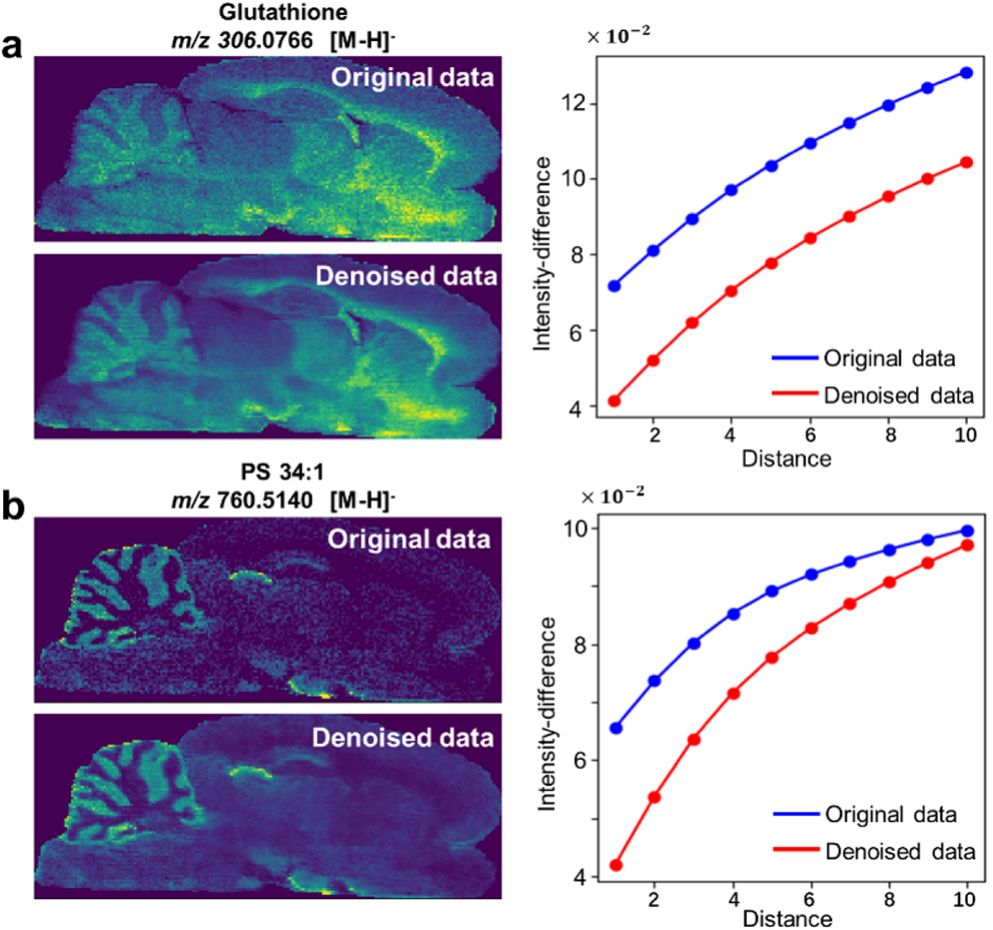
Results of original and denoised data of rat brain MSI acquired using DESI-MSI. (a) *m*/*z* 306.0766 Glutathione [M-H]^−^; (b) *m*/*z* 760.5140 PS 34:1 [M-H]^−^. The left panel displays the spatial distribution of the original ion and the corresponding denoised ion, while the right panel illustrates the intensity differences relative to the spatial distance.

## Conclusion

Enhancing the MSI data quality is a crucial preprocessing step that facilitates more effective mining and interpretation of the data. Computational strategies that directly apply data denoising techniques to MSI data offer a valuable alternative to or enhancement of traditional experimental methods. In this study, we propose a novel deep learning-based model, De-MSI, for denoising MSI data. The current results demonstrate that De-MSI outperforms other commonly used methods in effectively eliminating noise from original ion images on visual inspection and quantitative evaluation.

The benefits of the proposed De-MSI method can be attributed to the following factors: (1) Different from the commonly used methods that adopt a window-based spatial information reconstruction strategy and often produce the oversmoothed denoised images with poor high-frequency information, the proposed De-MSI method utilizes the U-Net architecture to effectively extract both global and local features from original ion images, resulting in the denoised images with rich high-frequency and low-frequency information. (2) By incorporating chemical prior knowledge from mass spectrometry, the pair of isotopic ions and monoisotopic ions are identified to create a reliable training data set to optimize the De-MSI model in a self-supervised manner, without making assumptions about the noise distribution. Consequently, compared to previous methods that often focus on refining the noise distribution within MSI data, De-MSI achieves better denoised images that more closely resemble those generated in real systems. (3) The proposed method can be effectively applied for data denoising on wide-range MSI data generated from various techniques, such as MALDI, DESI, or other ionization sources.

There are, however, the inclusion of MSI data across different modalities and resolutions to validate De-MSI is still limited. Applying De-MSI to other ionization sources, such as SIMS, which often produce super-resolution MSI data with a significant abundance of secondary ions, may further demonstrate its effectiveness in MSI data denoising. Furthermore, although the use of DeepION followed by manual inspection for the identification of monoisotopic-isotopic ion pairs effectively reduces isobaric interference, the intrinsic limitations of non high-resolution mass spectrometers may still have low-abundance contaminants to affect the denoising performance of De-MSI. Overall, our results demonstrate that isotopic pairs are relatively accessible from various MSI platforms and that a training set containing isotopic pairs equivalent to approximately 10–20% of the total detected ions is sufficient to achieve robust De-MSI model denoising. Future studies would incorporate MS/MS validation to further improve ion pairing accuracy and thereby enhance the denoising performance of the De-MSI model.

In summary, the present results highlight the significant potential of De-MSI in enhancing the quality of the MSI data. The proposed De-MSI method can be broadly applied to improve the quality of the high-resolution MSI data and those generated from various techniques. We expect the proposed De-MSI to emerge as a promising tool for facilitating the extensive applications of the MSI technique in single-cell metabolomics, biomarker discovery, and even metabolic flux analysis in the future.

## Supplementary Material



## Data Availability

The source code of the De-MSI model together with the data set for testing is available on https://github.com/gankLei-X/De-MSI.

## Notes

Spatial distribution of monotopic ions and isotopic ions in the MSI data set of mouse fetus (Figure S1); representative isotopic-monoisotopic ion pairs from the training data for MSI data set of mouse fetus (Figure S2); spatial distribution of original ion images alongside their corresponding denoised ion images generated using the De-MSI method on mouse fetus data set (Figure S3); spatial distribution and corresponding PSRN and SSIM values for simulated ion image, denoised ion image generated using Gaussian filtering, Wavelet denoising, De-MSI model, and the ground truth on mouse fetus data set (Figure S4); representative isotopic-monoisotopic ion pairs from the training data for MSI data set of mouse brain (Figure S5); optical imaging of high-resolution mouse brain data set (Figure S6); comparison of signal intensities between original and denoised data on MSI data set of mouse brain, with the true signal intensities from the original data serving as the reference (Figure S7); spatial distribution for original ion image, denoised ion image generated using Gaussian filtering, Wavelet denoising on mouse brain data set (Figure S8); spatial distribution of original image and denoised image within MSI data set of mouse brain acquired using MALDI at a pixel size of 5 μm (Figure S9); representative isotopic-monoisotopic ion pairs from the training data for MSI data set of rat brain (Figure S10); spatial distribution of original image and denoised image within MSI data set of rat brain acquired using DESI (Figure S11); data distribution statistics for the three MSI data sets (Table S1); detailed protocols for sample preparation and data acquisition (Material S1); detailed workflow for the identification of monoisotopic-isotopic ion pairs (Material S2); quantitative evaluation (Material S3) (PDF)

## References

[1] Xie Y. R., Castro D. C., Rubakhin S. S., Trinklein T. J., Sweedler J. V., Lam F. (2024). Multiscale biochemical mapping of
the brain through deep-learning-enhanced high-throughput mass spectrometry. Nat. Methods.

[2] Doerr A. (2018). Mass spectrometry
imaging takes off. Nat. Methods.

[3] Zhang J., Due Q. Q., Song X. W., Gao S. S., Pang X. C., Li Y., Zhang R. P., Abliz Z., He J. M. (2020). Evaluation of the
tumor-targeting efficiency and intratumor heterogeneity of anticancer
drugs using quantitative mass spectrometry imaging. Theranostics.

[4] Diao X., Xie C. Y., Xie G. S., Song Y. Y., Liang Y. S., Li R. J., Dong C., Zhu L., Wang J. N., Cai Z. W. (2022). Mass spectrometry imaging revealed sulfatides depletion
in brain tissues of rats exposed in real air with high fine particulate
matter. Environ. Sci. Technol. Lett..

[5] Djambazova K. V., Van Ardenne J. M., Spraggins J. M. (2023). Advances in imaging mass spectrometry
for biomedical and clinical research. Trac-Trend.
Anal. Chem..

[6] Verbeeck N. R. M., Caprioli R., de Plas R. (2020). Unsupervised
machine learning for
exploratory data analysis in imaging mass spectrometry. Mass Spectrom. Rev..

[7] Dexter A., Race A. M., Styles I. B., Bunch J. (2016). Testing for multivariate
normality in mass spectrometry imaging data: A robust statistical
approach for clustering evaluation and the generation of synthetic
mass spectrometry imaging data sets. Analy.
Chem..

[8] Guo X., Wang X., Tian C. Y., Dai Z. J., Zhao Z., Duan Y. (2023). Development of mass spectrometry imaging techniques and its latest
applications. Talanta.

[9] Guo L., Hu Z. X., Zhao C., Xu X. N., Wang S. J., Xu J. J., Dong J. Y., Cai Z. W. (2021). Data Filtering and
Its Prioritization in Pipelines for Spatial Segmentation of Mass Spectrometry
Imaging. Anal. Chem..

[10] Tyler B. J., Kassenbohmer R., Peterson R. E., Nguyen D. T., Freitag M., Glorius F., Ravoo B. J., Arlinghaus H. F. (2022). Denoising
of mass spectrometry images via inverse maximum signal factors analysis. Analy. Chem..

[11] Abdelmoula W. M., Lopez B. G., Randall E. C., Kapur T., Sarkaria J. N., White F. M., Agar J. N., Wells W. M., Agar N. Y. R. (2021). Peak
learning of mass spectrometry imaging data using artificial neural
networks. Nat. Commun..

[12] Wickes B. T., Kim Y. M., Castner D. G. (2003). Denoising and multivariate
analysis
of time-of-flight SIMS images. Surf. Interface
Anal..

[13] Tian C., Fei L. K., Zheng W. X., Xu Y., Zuo W. M., Lin C. W. (2020). Deep learning on image denoising: An overview. Neural Networks.

[14] Izadi S., Sutton D., Hamarneh G. (2023). Image denoising in
the deep learning
era. Artif. Intell. Rev..

[15] Qiao C., Li D., Liu Y., Zhang S. W., Liu K., Liu C., Guo Y. T., Jiang T., Fang C. Y., Li N. (2023). Rationalized
deep learning super-resolution microscopy for sustained
live imaging of rapid subcellular processes. Nat. Biotechnol..

[16] Zhang K., Zuo W. M., Chen Y. J., Meng D. Y., Zhang L. (2017). Beyond a Gaussian
denoiser: Residual learning of deep CNN for image denoising. IEEE. T. Image. Process..

[17] Guo L., Xie C. Y., Miao R., Xu J. J., Fang J. C., Wang X. X., Liu W. P., Liao X. W., Wang J. N., Dong J. Y. (2024). DeepION:
A deep learning-based low-dimensional representation
model of ion images for mass spectrometry imaging. Anal. Chem..

[18] Palmer A., Phapale P., Chernyavsky I., Lavigne R., Fay D., Tarasov A., Kovalev V., Fuchser J., Nikolenko S., Pineau (2017). C.FDR-controlled
metabolite annotation for high-resolution imaging mass spectrometry. Nat. Methods.

[19] Sementé L., Baquer G., García-Altares M., Correig-Blanchar X., Ràfols P. (2021). rMSIannotation: A peak annotation tool for mass spectrometry
imaging based on the analysis of isotopic intensity ratios. Anal. Chim. Acta.

[20] Ronneberger, O. ; Fischer, P. ; Brox, T. U-net: Convolutional networks for biomedical image segmentation. In 18th International Conference on Medical Image Computing and Computer-Assisted Intervention; Springer, 2015.

[21] Falk T., Mai D., Bensch R., Cicek C., Abdulkadir A., Marrakchi Y., Böhm A., Deubner J., Jäckel Z., Seiwald K. (2019). U-Net: deep learning for cell counting, detection,
and morphometry. Nat. Methods.

[22] Kingma D. P., Ba J. (2014). Adam: A method for stochastic optimization. arXiv.

[23] Wüllems K., Zurowietz A., Zurowietz M., Schneider R., Bednarz H., Niehaus K., Nattkemper T. W. (2021). Fast visual
exploration of mass spectrometry images with interactive dynamic spectral
similarity pseudocoloring. Sci. Rep..

[24] Gardner W., Maliki R., Cutts S. M., Muir B. W., Ballabio D., Winkler D. A., Pigram P. J. (2020). Self-organizing
map and relational
perspective mapping for the accurate visualization of high-dimensional
hyperspectral data. Anal. Chem..

[25] Chen R., Xu J. S., Wang B. Q., Ding Y., Abdulla A., Li Y. Y., Jiang L., Ding X. T. (2024). SpiDe-Sr: blind
super-resolution network for precise cell segmentation and clustering
in spatial proteomics imaging. Nat. Commun..

[26] Deepaisarn S., Tar P. D., Thacker N. A., Seepujak A., McMahon A. W. (2018). Quantifying
biological samples using Linear Poisson Independent Component Analysis
for MALDI-ToF mass spectra. Bioinformatics.

[27] Keenan M. R., Trindade G. F., Pirkl A., Newell C. L., Jin Y., Aizikov K., Dannhorn A., Zhang J., Matjačić L., Arlinghaus H. (2025). Orbitrap noise structure and method for noise
unbiased multivariate analysis. Nat. Commun..

[28] Taylor S., Ponzini M., Wilson M., Kim K. (2022). Comparison of imputation
and imputation-free methods for statistical analysis of mass spectrometry
data with missing data. Briefings Bioinf..

[29] Horé, A. ; Ziou, D. Image Quality Metrics: PSNR vs. SSIM. In 20th International Conference On Pattern Recognition; IEEE, 2010.

[30] Wang Z., Bovik A. C., Sheikh H. R., Simoncelli E. P. (2004). Image quality
assessment: from error visibility to structural similarity. IEEE. T. Image. Process..

[31] Zhang H., Ding L., Hu A., Shi X. D., Huang P. H., Lu H. Y., Tillberg P. W., Wang M. C., Li L. J. (2025). TEMI: tissue-expansion
mass-spectrometry imaging. Nat. Methods.

[32] Hung Y. L. W., Xie C. Y., Wang J. N., Diao X., Li R. X., Wang X. X., Qiu S. L., Fang J. C., Cai Z. W. (2024). Expansion
strategy-driven micron-level resolution mass spectrometry imaging
of lipids in mouse brain iissue. CCS Chem..

[33] Ma S. Y., Leng Y. X., Li X. P., Meng Y. F., Yin Z. B., Hang W. (2023). High spatial
resolution mass spectrometry imaging for spatial metabolomics:
Advances, challenges, and future perspectives. Trac-Trend. Anal. Chem..

[34] Wang Q. X., Ding S. L., Li Y., Royall J., Feng D., Lesnar P., Graddis N., Naeemi M., Facer B., Ho A., Dolbeare T., Blanchard B., Dee N., Wakeman W., Hirokawa K. E., Szafer A., Sunkin S. M., Oh S. W., Bernard A., Phillips J. W., Hawrylycz M., Koch C., Zeng H. K., Harris J. A., Ng L. (2020). The Allen
Mouse Brain Common Coordinate Framework: A 3D Reference Atlas. Cell.

[35] Kleven H., Bjerke I. E., Clascá F., Groenewegen H. J., Bjaalie J. G., Leergaard T. B. (2023). Waxholm
Space atlas of the rat brain:
a 3D atlas supporting data analysis and integration. Nat. Methods.

