# Supplementary material for: De-MSI: A Deep Learning-Based Data Denoising Method to Enhance Mass Spectrometry Imaging by Leveraging the Chemical Prior Knowledge
Source: Anal Chem. 2025 Sep 8;97(37):20201–8. doi: 10.1021/acs.analchem.5c02946 (PMC12461676; doi:10.1021/acs.analchem.5c02946)
Supplement: Supplementary file 1 [file ac5c02946_si_001.pdf]

Supplementary Materials

De-MSI: a Deep Learning-based Data Denoising Method to Enhance Mass Spectrometry Imaging by Leveraging the Chemical Prior Knowledge

Lei Guo[1,2], Chengyi Xie[2], Xin Diao[2], Thomas Ka Yam Lam[2], Yanhui Zhong[2], Yanyan Chen[2], Jingjing Xu[3], Xiangnan Xu[4], Xiangyu Zhu[2], Zhuang Xiong[1], Shangyi Luo[1], Jianing Wang[2,5], Jiyang Dong[3]*, Zongwei Cai[2,6]*

[1] Interdisciplinary Institute for Medical Engineering, Fuzhou University, Fuzhou 350108, China

[2] State Key Laboratory of Environmental and Biological Analysis, Hong Kong Baptist University, Hong Kong SAR 999077, China

[3] Department of Electronic Science, National Institute for Data Science in Health and Medicine, Xiamen University, Xiamen 36100, China

[4] School of Business and Economics, Humboldt-Universitat zu Berlin, Berlin 10099, Germany

[5] School of Marine Science and Engineering, Hainan University, Haikou 570228, China

[6] College of Science, Eastern Institute of Technology, Ningbo 315200, China

*Corresponding Author(s):

Zongwei Cai: zwcai@hkbu.edu.hk

Jiyang Dong: jydong@xmu.edu.cn

CONTENT

1. Supplementary Figures S1

Figure S1. Spatial distribution of monotopic ions and isotopic ions in the MSI dataset of mouse fetus S1

Figure S2. Representative isotopic-monoisotopic ion pairs from the training data for MSI dataset of mouse fetus. S2

Figure S3. Spatial distribution of original ion images alongside their corresponding denoised ion images generated using the De-MSI method on mouse fetus dataset. S3

Figure S4. Spatial distribution and corresponding PSRN and SSIM values for simulated ion image, denoised ion image generated using Gaussian Filtering, Wavelet denoising, De-MSI model, and the ground truth on mouse fetus dataset. S4

Figure S5. Representative isotopic-monoisotopic ion pairs from the training data for MSI dataset of mouse brain. S5

Figure S6. Optical imaging of high-resolution mouse brain dataset. S6

Figure S7. Comparison of signal intensities between original and denoised data on MSI dataset of mouse brain, with the true signal intensities from the original data serving as the reference. S7

Figure S8. Spatial distribution for original ion image, denoised ion image generated using Gaussian Filtering, Wavelet denoising on mouse brain dataset S8

Figure S9. Spatial distribution of original image and denoised image within MSI dataset of mouse brain acquired using MALDI-MSI at a pixel size of 5 μm. S9

Figure S10. Representative isotopic-monoisotopic ion pairs from the training data for MSI dataset of rat brain. S10

Figure S11. Spatial distribution of original image and denoised image within MSI dataset of rat brain acquired using DESI-MSI. S11

2. Supplementary Tables S12

Table S1 Data distribution statistics for the three MSI datasets................................. S12

3. Supplementary Materials S13

Material S1 Detailed protocols for sample preparation and data acquisition S13

Material S2 Detailed workflow for the identification of monoisotopic-isotopic ion pairs S14

Material S3 Quantitative Evaluation.. S15

References Cited in Supporting Information S16

1. Supplementary Figures

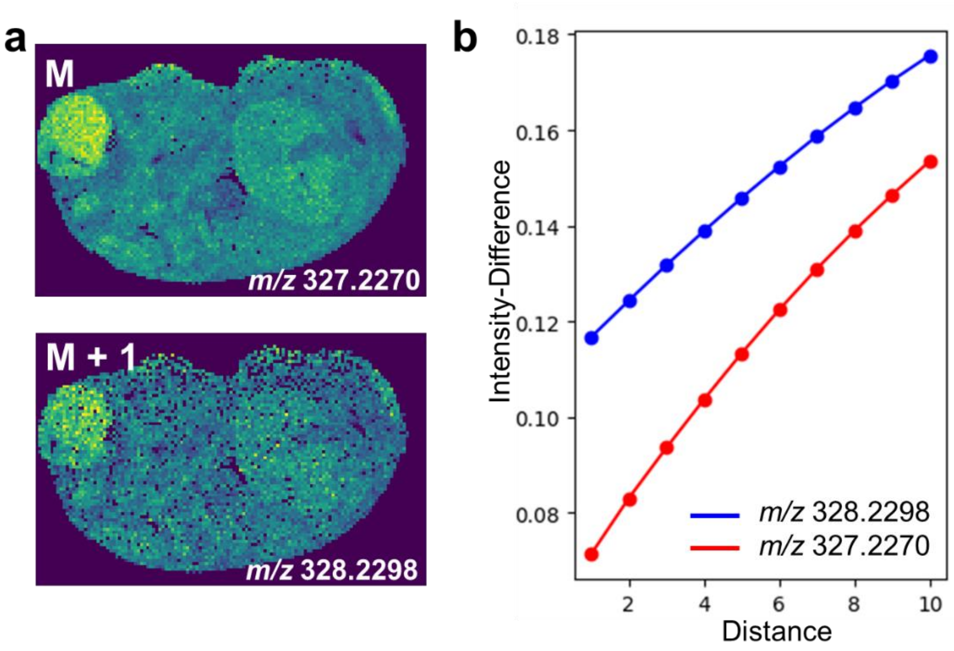

Figure S1. Spatial distribution of monotopic ions and isotopic ions in the MSI dataset of mouse fetus. (a) Displays the ion images at *m/z* 327.2270, *m/z* 328.2298. (b) Shows the intensity differences of *m/z* 327.2270 and *m/z* 328.229 relative to spatial distance. In fetal tissue, metabolites are expected to have similar expressions in adjacent pixels. The monoisotopic ion exhibits the lowest intensity differences at varying distances, indicating higher data quality than isotopic ion.

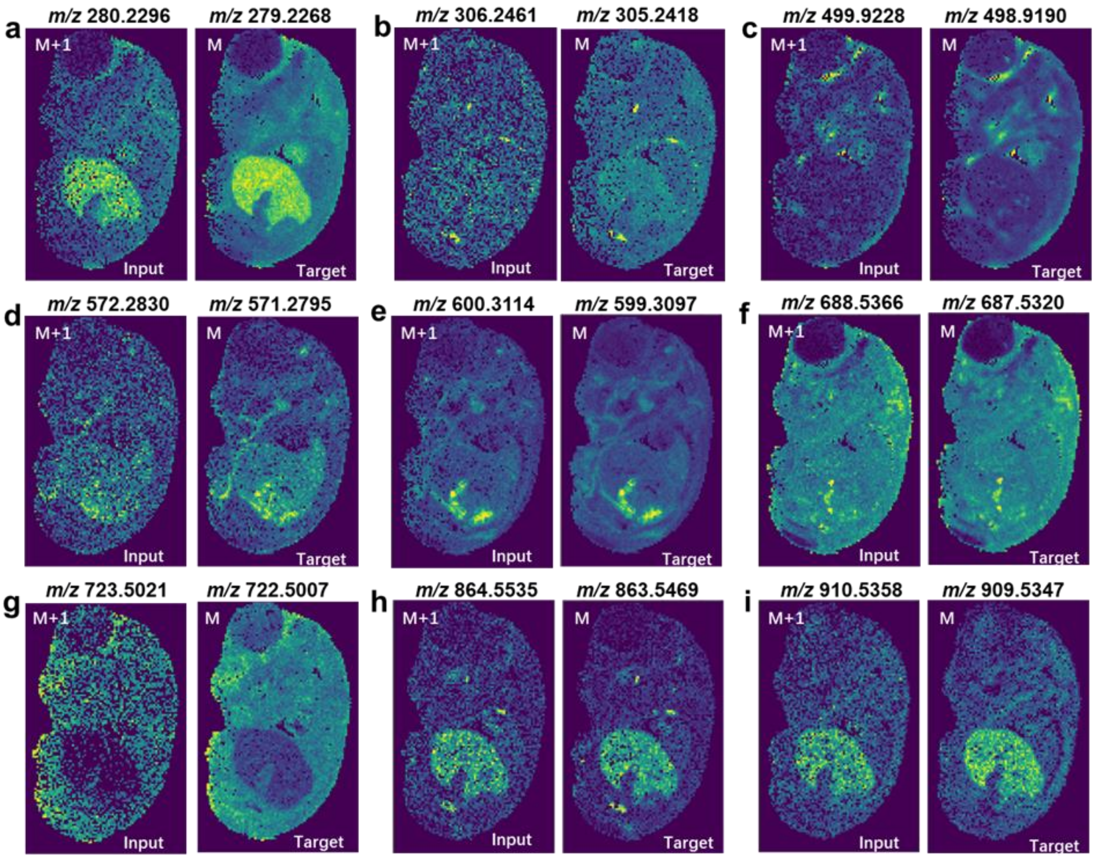

Figure S2. Representative isotopic-monoisotopic ion pairs from the training data for MSI dataset of mouse fetus. (a) *m/z* 280.2296 and *m/z* 279.2268; (b) *m/z* 306.2461 and *m/z* 305.2418; (c) *m/z* 499.9228 and *m/z* 498.9190; (d) *m/z* 572.2830 and *m/z* 571.2795; (e) *m/z* 600.3114 and *m/z* 599.3097; (f) *m/z* 688.5366 and *m/z* 687.5320; (g) *m/z* 723.5021 and *m/z* 722.5007; (h) *m/z* 864.5535 and *m/z* 863.5469; (i) *m/z* 910.5358 and *m/z* 909.5347.

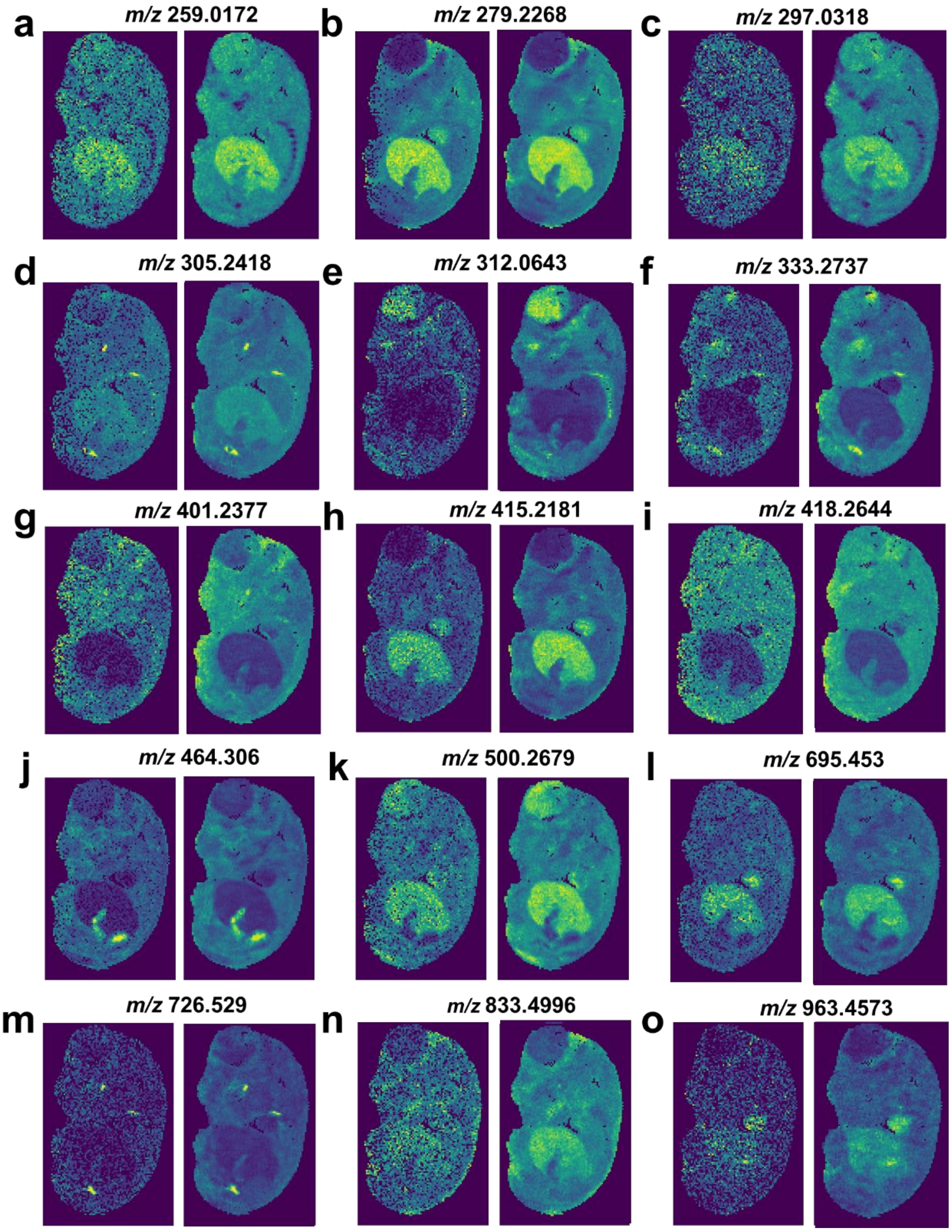

Figure S3. Spatial distribution of original ion images alongside their corresponding denoised ion images generated using the De-MSI method on mouse fetus dataset. (a) *m/z* 259.0172; (b) *m/z* 279.2268; (c) *m/z* 297.0318; (d) *m/z* 305.2418; (e) *m/z* 312.0643; (f) *m/z* 333.2737; (g) *m/z* 401.2377; (h) *m/z* 415.2181; (i) *m/z* 418.2644; (j) *m/z* 464.3060; (k) *m/z* 500.2679; (l) *m/z* 695.4530; (m) *m/z* 726.5290; (n) *m/z* 833.4996; (o) *m/z* 963.4573. Note that there are no isotopic ions included here.

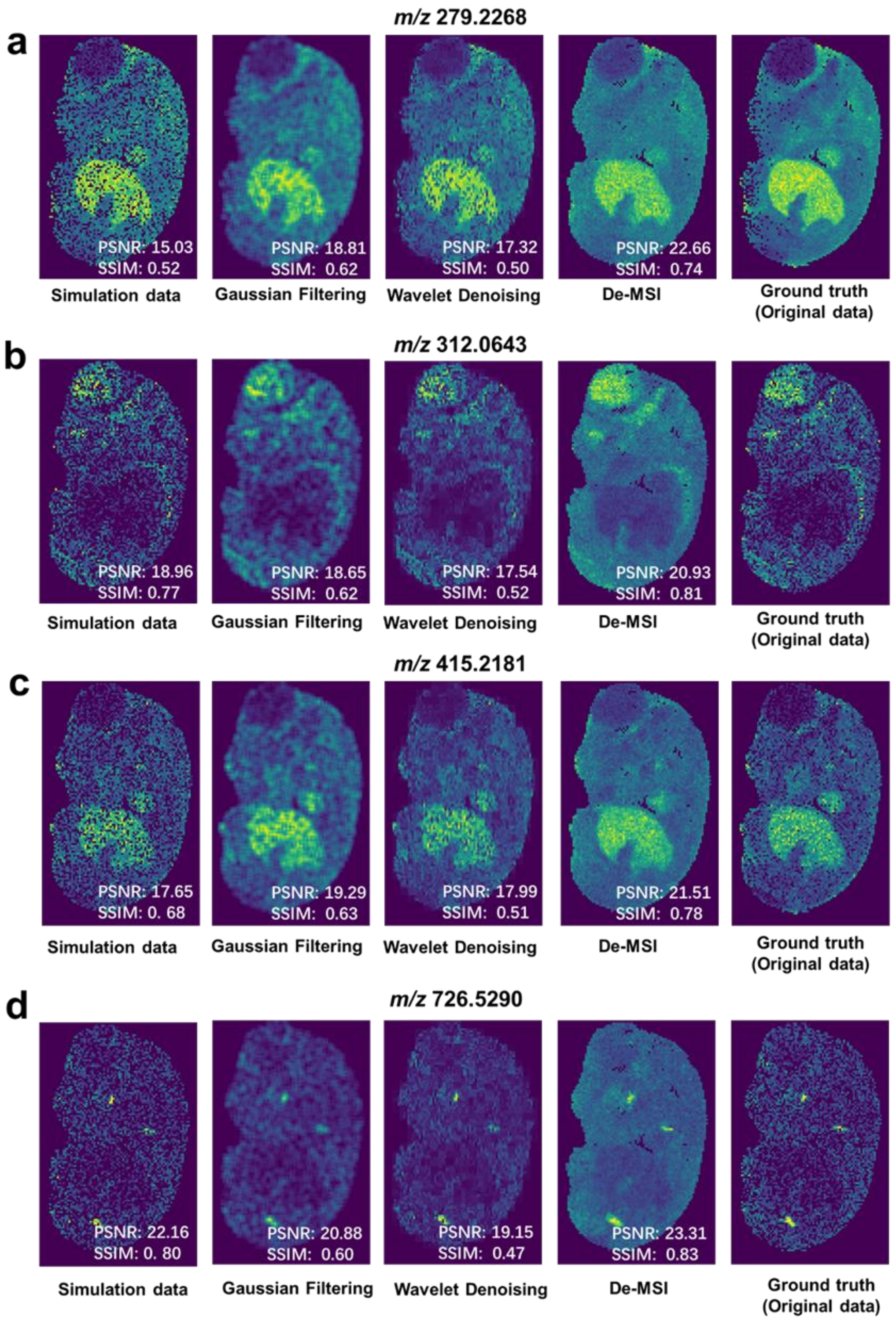

Figure S4. Spatial distribution and corresponding PSRN and SSIM values for simulated ion image, denoised ion image generated using Gaussian Filtering, Wavelet denoising, De-MSI model, and the ground truth on mouse fetus dataset. (a) *m/z* 279.2268; (b) *m/z* 312.0643; (c) *m/z* 415.2181; (d) *m/z* 726.5290. Note that there are no isotopic ions included here.

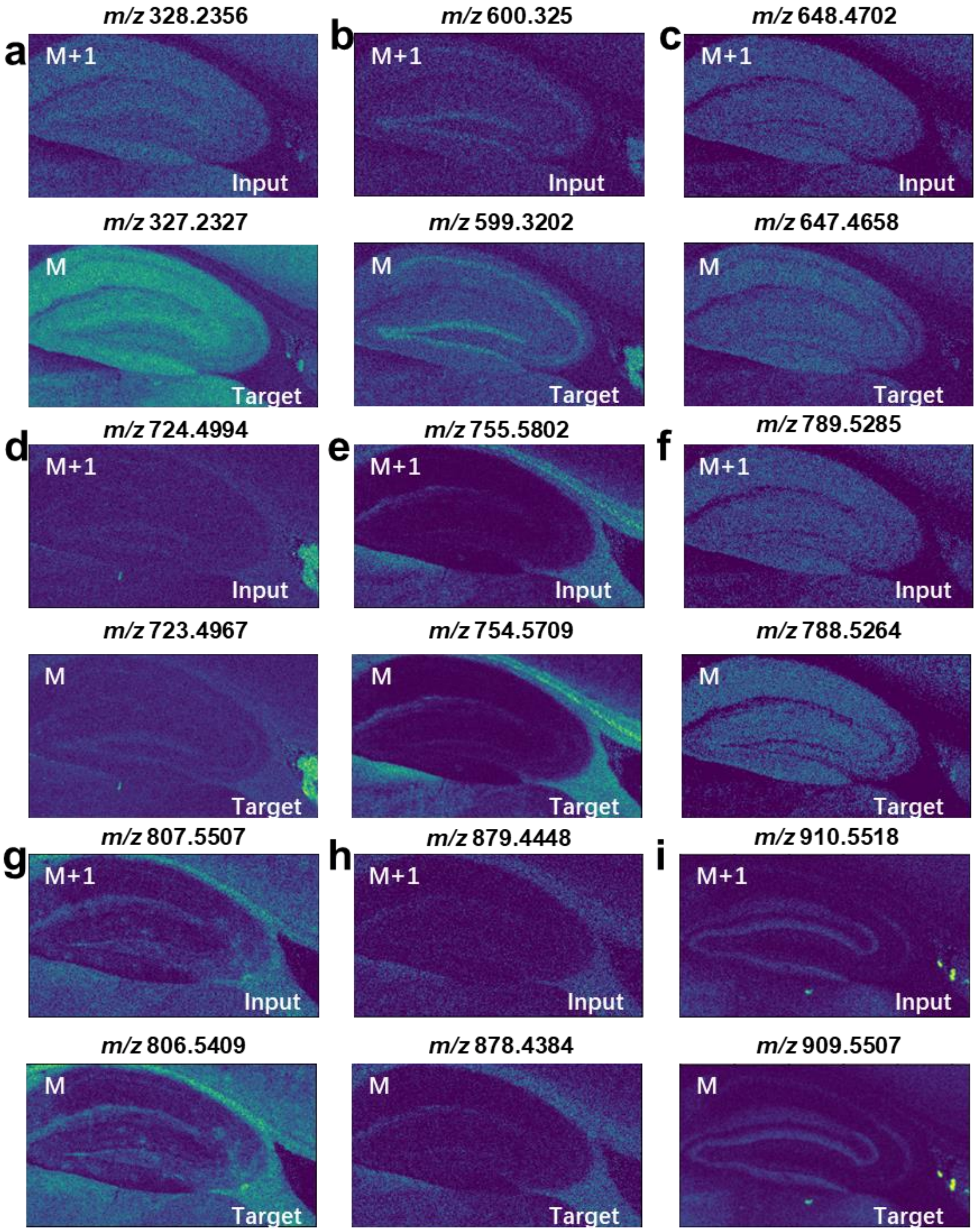

Figure S5. Representative isotopic-monoisotopic ion pairs from the training data for MSI dataset of mouse brain. (a) *m/z* 328.2356 and *m/z* 327.2327; (b) *m/z* 600.3250 and *m/z* 599.3202; (c) *m/z* 648.4702 and *m/z* 647.4658; (d) *m/z* 724.4994 and *m/z* 723.4967; (e) *m/z* 755.5802 and *m/z* 754.5709; (f) *m/z* 789.5285 and *m/z* 788.5264; (g) *m/z* 807.5507 and *m/z* 806.5409; (h) *m/z* 879.4448 and *m/z* 878.4384; (i) *m/z* 910.5518 and *m/z* 909.5507.

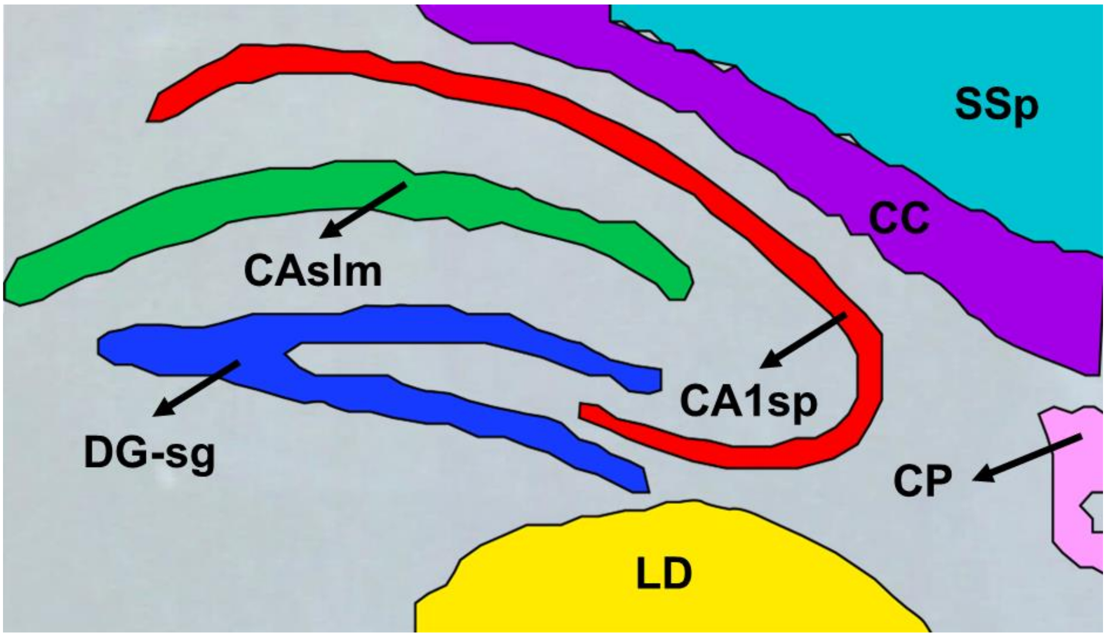

Figure S6. Optical imaging of high-resolution mouse brain dataset. Seven regions including the DG-sg, CAslm, CA1sp, CC, SSp, CP and LD are highlighted in blue, green, red, purple, light blue, pink and yellow, respectively. DG-sg: granule cell layer; CAslm: stratum lacunosum-moleculare; CA1sp: pyramidal layer; CC: corpus callosum; SSp: primary somatosensory area; CP: caudoputamen; LD: lateral dorsal nucleus of thalamus.

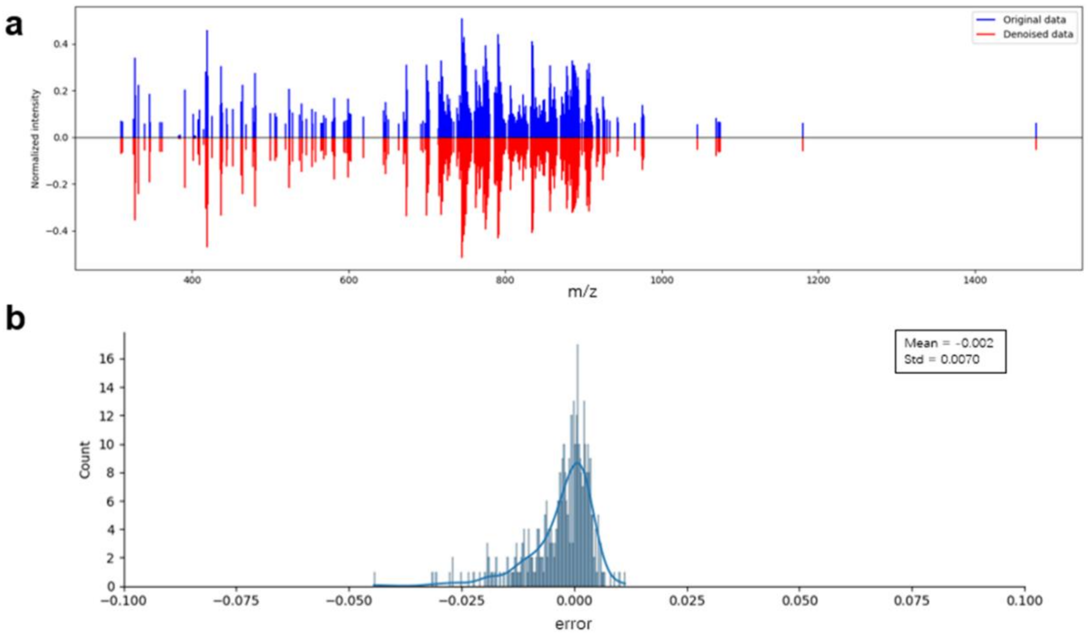

Figure S7. Comparison of signal intensities between original and denoised data on MSI dataset of mouse brain, with the true signal intensities from the original data serving as the reference. (a) Mean spectra of both original and denoised datasets; (b) Distribution of errors across all true data points, visualized using sns.displot. The resulting error distribution (denoised vs. original) is tightly centered around zero (mean error: -0.0027 ± 0.0070 SD), indicating no significant deviation within regions corresponding to true biological signals.

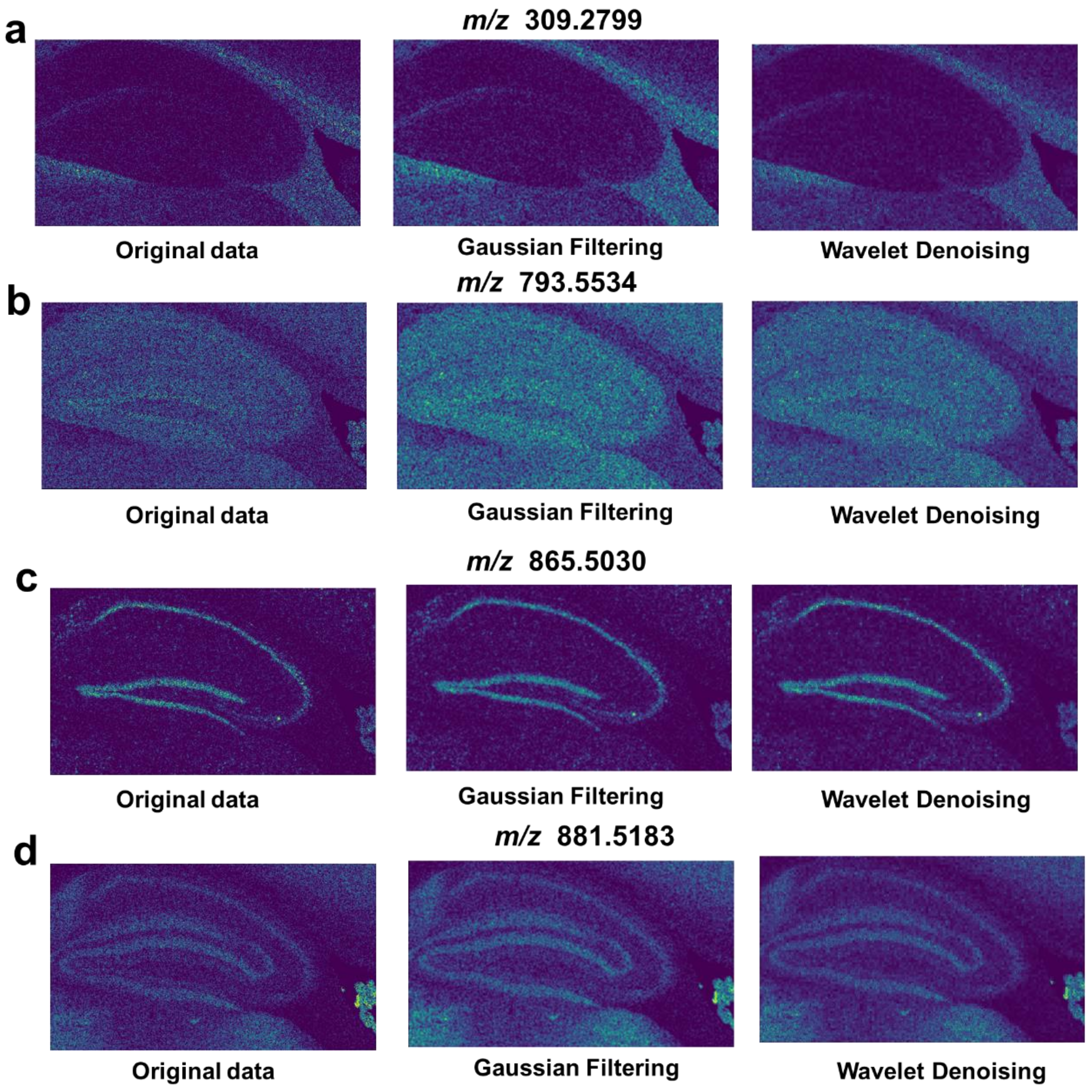

Figure S8. Spatial distribution for original ion image, denoised ion image generated using Gaussian Filtering, Wavelet denoising on mouse brain dataset. (a) *m/z* 309.2799; (b) *m/z* 793.5534; (c) *m/z* 865.5030; (d) *m/z* 881.5183.

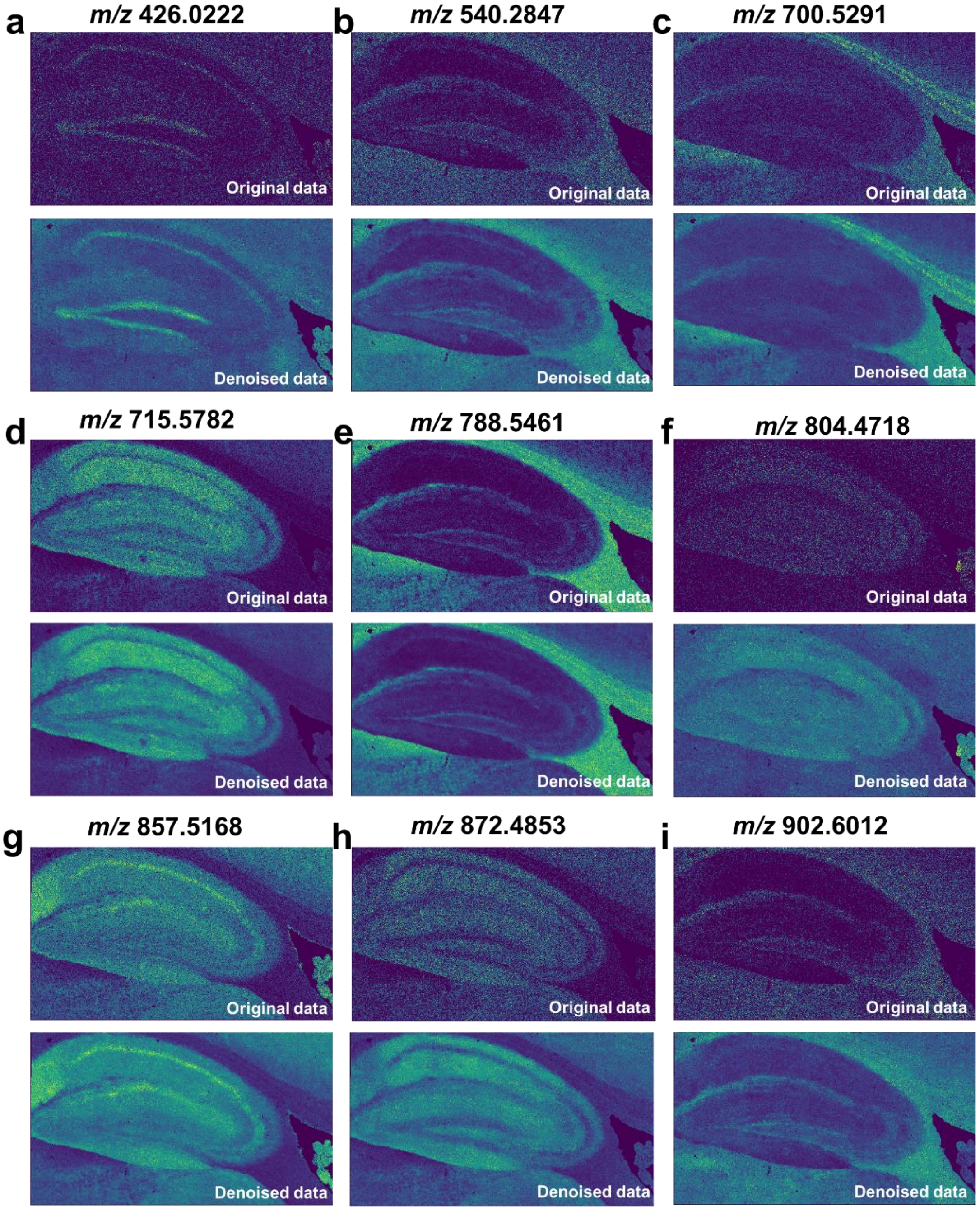

Figure S9. Spatial distribution of original image and denoised image within MSI dataset of mouse brain acquired using MALDI-MSI at a pixel size of 5 μm. (a) *m/z* 426.0222; (b) *m/z* 540.2847; (c) *m/z* 700.5291; (d) *m/z* 715.5782; (e) *m/z* 788.5461; (f) *m/z* 804.4718; (g) *m/z* 857.5168; (h) *m/z* 872.4853; (i) *m/z* 902.6012.

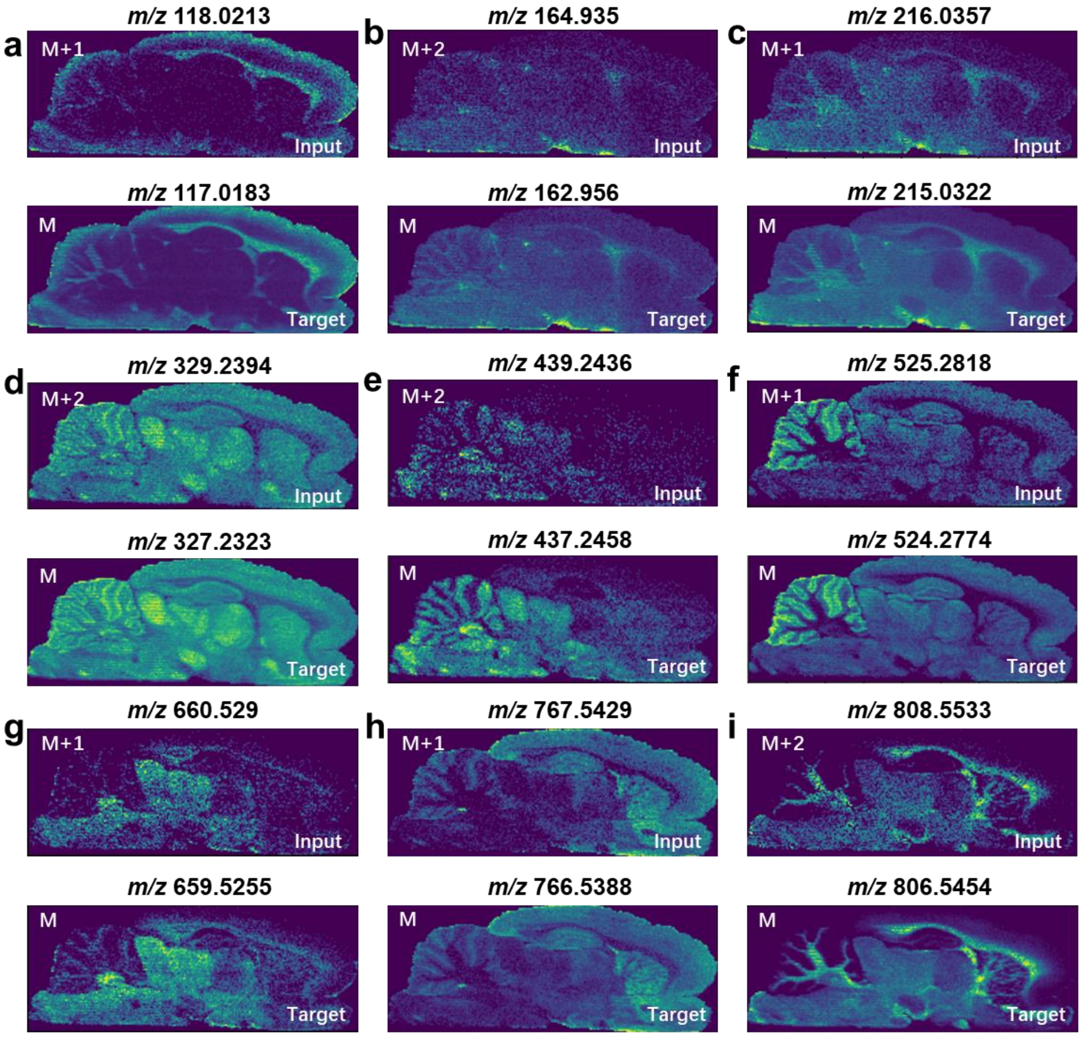

Figure S10. Representative isotopic-monoisotopic ion pairs from the training data for MSI dataset of rat brain. (a) *m/z* 118.0213 and *m/z* 117.0183; (b) *m/z* 164.9350 and *m/z* 162.9560; (c) *m/z* 216.0357 and *m/z* 215.0322; (d) *m/z* 329.2394 and *m/z* 327.2323; (e) *m/z* 439.2436 and *m/z* 437.2458; (f) *m/z* 525.2818 and *m/z* 524.2774; (g) *m/z* 660.5290 and *m/z* 659.5255; (h) *m/z* 767.5429 and *m/z* 766.5388; (i) *m/z* 808.5533 and *m/z* 806.5454.

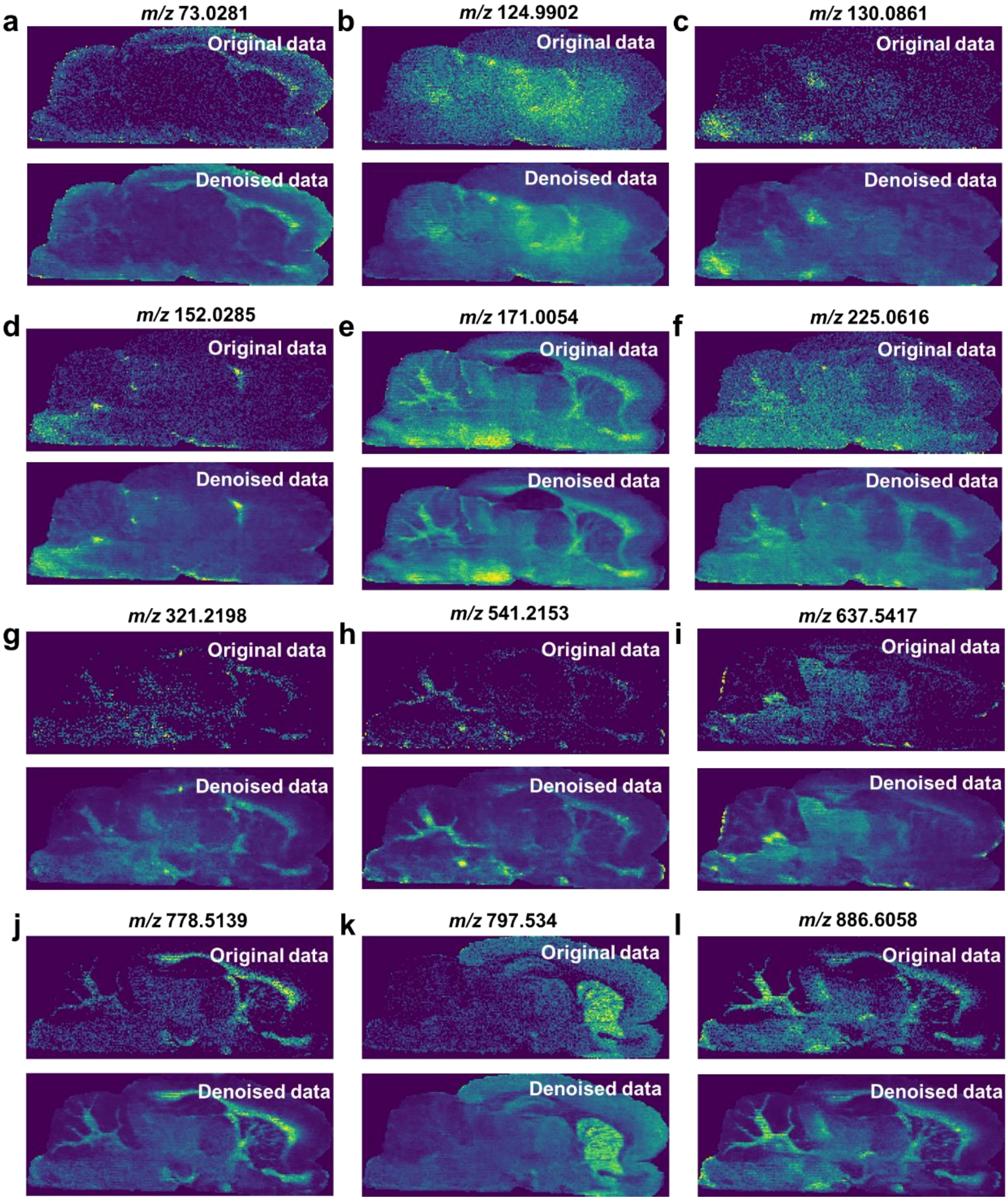

Figure S11. Spatial distribution of original image and denoised image within MSI dataset of rat brain acquired using DESI-MSI. (a) *m/z* 73.0281; (b) *m/z* 124.9902; (c) *m/z* 130.0861; (d) *m/z* 152.0285; (e) *m/z* 171.0054; (f) *m/z* 225.0616; (g) *m/z* 321.2198; (h) *m/z* 541.2153; (i) *m/z* 637.5417; (j) *m/z* 778.5139; (k) *m/z* 797.5340; (l) *m/z* 886.6058. Note that there are no isotopic ions included here.

2. Supplementary Tables

Table S1 Data distribution statistics for the three MSI datasets

Tissue	Ionization Sources	Dataset Details	Monoisotopic-isotopic Ion Pair Number
Mouse fetus	MALDI-MSI	82×127 pixels with 586 *m/z* features, measured at 100 μm	103
Mouse brain	MALDI-MSI	305×543 pixels with 381 *m/z* features, measured at 5 μm	129
Rat brain	DESI-MSI	215×86 pixels with 901 *m/z* features, measured at 100 μm	117

3. Supplementary Materials

Material S1 Detailed protocols for sample preparation and data acquisition

A. Sample preparation

The mice and rats are maintained in a clean setting with individually ventilated cages, adhering to a 12-hour cycle of light and darkness, at a temperature of 22°C and 45% relative humidity. They are provided with unlimited access to food and water under standard conditions. Entire GD 14.5 mouse fetus, mouse brain, and rat brain are collected, and immediately preserved at -80°C for subsequent experiments.

B. Data Acquisition

In this study, MALDI-MSI and DESI-MSI techniques are used to analyze tissue sections. Specifically, these tissues are cryosectioned to 12 μm using a CryoStar Nx70 cryostat (Thermal Fisher Scientific, Germany). For MALDI-MSI experiments, slices were placed on ITO-coated glass slides (Delta Technologies, USA) while normal glass slides (Epredia, USA) were used for DESI-MSI experiments. 1,5-diaminonaphthalene (DAN) matrix (5 mg/mL in MeOH) was freshly prepared, and five layers were applied via a custom electrospray system[1]. Data from the mouse fetus and mouse brain were acquired using the timsTOF flex MALDI-2 instrument (Bruker Daltonics, Germany) in negative ion mode. The mouse fetus data was collected at a lateral resolution of 100 μm with a mass range of 300-1400 m/z, while the mouse brain data is captured using the highest lateral resolution of timsTOF flex (i.e. 5 μm) with a mass range of 200-1000 m/z. For DESI-MSI experiments, rat brain tissue was analyzed using AFADESI (Beijing Victor Technology Co., China) equipped with a Q Exactive HF mass spectrometer in negative ion mode. ACN/H2O (8:2, 0.1% formic acid) was used as a solvent at a flow rate of 5 μL/min. Data is acquired at a mass range of 70–1000 m/z, scanning at 0.4 mm/s horizontally and stepping 100 μm vertically.

Material S2 Detailed workflow for the identification of monoisotopic-isotopic ion pairs

To ensure reliable training pairs for both MALDI-TimsToF (mouse fetus, mouse brain) and DESI-Orbitrap (rat brain) datasets, we implemented a dual-step strategy:

- **Step 1:** Automated pairing of monoisotopic-isotopic ions using DeepION model, which leverages spatial-spectral correlations to minimize interference.
- **Step 2:** Manual curation based on spatial concordance: Candidate pairs were retained only if they exhibited consistent spatial distributions (Pearson's $r > 0.85$), effectively excluding ions with significant isobaric contamination.

Material S3 Quantitative Evaluation

In this study, we employ Poisson noise and random missing to simulate the noise typically encountered during data acquisition. This methodology is supported by previous research indicating that both noise and signal in MSI data follow Poisson statistical distributions[2], [3], with a notable occurrence of zero values frequently observed in standard spectra[4]. Then, to add noise into the original MSI data, we design a two-step process: (1) For each pixel in the ion image, a random intensity is generated to replace the original intensity, following a Poisson distribution with the pixel value as the mean. It is implemented using the `poisson.rvs` function from the SciPy Python package; (2) Randomly set 20% of the pixel signals in each ion image to zero. Finally, two metrics including peak signal-to-noise ratio (PSNR) and structural similarity index measure (SSIM) are employed to evaluate the difference between original data and denoised data. Note that missing values in the original data are not included in the quantitative evaluation. Specifically, PSNR focusing on pixel-level differences, it is calculated as follows:

$$PSNR = 10 \cdot log_{10}(\frac{MAX^2}{MSE}) \tag{1}$$

where MAX is the max intensity in ion image. MSE is calculated as:

$$MSE = \frac{1}{XY}\sum_{i=1}^{X}\sum_{i=1}^{Y}(I(i,j) - K(i,j))^2 \tag{2}$$

where the X and Y are the horizontal and vertical pixel counts, $I(i,j)$ and $K(i,j)$ are the pixel intensity of original image and the processed image, respectively. SSIM evaluates the perceptual quality of images by analyzing changes in structural information, luminance, and contrast. It is calculated as follows:

$$SSIM = \frac{(2u_I u_K + C_1)(2\sigma_{IK} + C_2)}{(u_I^2 + u_K^2 + C_1)(\sigma_I^2 + \sigma_K^2 + C_2)} \tag{3}$$

where u_I and u_K are mean intensities of original ion image I and processed image K, σ_I^2 and σ_K^2 are the variances of I and K, C_1 and C_2 are constants which are defined as:

$$C_1 = (K_1 L)^2 \qquad C_2 = (K_2 L)^2 \tag{4}$$

where L is the dynamic range of the pixel values, K_1 and K_2 are constants set to

default values of 0.01 and 0.03, respectively.

References Cited in Supporting Information

1. Xie, C. Y.; Chen, Y. Y.; Wang, X. X.; Song, Y. Y.; Shen, Y. T.; Diao, X.; Zhu, L.; Wang, J. N.; Cai, Z. W. Chiral derivatization-enabled discrimination and on-tissue detection of proteinogenic amino acids by ion mobility mass spectrometry. *Chem. Sci.* **2022**, 13(47), 14114-14123.
2. Deepaisarn, S.; Tar, P. D.; Thacker, N. A.; Seepujak, A.; McMahon, A. W. Quantifying biological samples using Linear Poisson Independent Component Analysis for MALDI-ToF mass spectra. *Bioinformatics.,* **2018,** 34(6), 1001-1008.
3. Keenan, M. R; Trindade, G. F; Pirkl, A.; Newell, C. L.; Jin, Y.; Aizikov, K.; Dannhorn, A.; Zhang, J.; Matjačić, L.; Arlinghaus, H.; Eyres, A.; Havelund, R.; Goodwin, R. J. A.; Takats, Z.; Bunch, J.; Gould, A. P.; Makarov, A.; Gilmore, I. S. Orbitrap noise structure and method for noise unbiased multivariate analysis. *Nat. Commun.*; **2025**, 16(1), 6398.
4. Taylor, S.; Ponzini, M.; Wilson, M.; Kim, K. Comparison of imputation and imputation-free methods for statistical analysis of mass spectrometry data with missing data. *Brief. Bioinform.*, **2022**, 23(1), bbab353.
